# Supplementary material for: Impact of antibiotics on off-target infant gut microbiota and resistance genes in cohort studies
Source: Pediatr Res. 2022 May 14;92(6):1757–66. doi: 10.1038/s41390-022-02104-w (PMC9659678; doi:10.1038/s41390-022-02104-w)
Supplement: Supplementary file 1 — Supplementary Figures and Notes [file 41390_2022_2104_MOESM1_ESM.pdf]

Impact of antibiotics to off-target infant gut microbiota and resistance genes in cohort studies

Rebecca M. Lebeaux, Juliette C. Madan, Quang P. Nguyen, Modupe O. Coker, Erika F. Dade, Yuka Moroishi, Thomas J. Palys, Benjamin D. Ross, Melinda M. Pettigrew, Hilary G. Morrison, Margaret R. Karagas, Anne G. Hoen

Table of Contents

Supplemental Figure:
Figure S1: Description of study inclusion criteria and schema for infants………………..……...2
Figure S2: Correlation analysis of species and ARGs by antibiotic exposure and day care attendance in the first year of life…………………………………………………………………3

Alpha and beta diversity analysis ……………….……………………………………………...5

Supplemental Notes:
Antibiotic exposure classification and sensitivity analyses – methods……………………....16
Antibiotic exposure classification and sensitivity analyses – results………………………...20
Covariates………………………………………………………………………………….24
Stool microbiome assessment……………………………………………………………..24
Statistical analyses…………………………………………………………………………...27
Difference-in-differences assumptions and sensitivity analyses………………..……………30
References…………………………………………………………………….........34

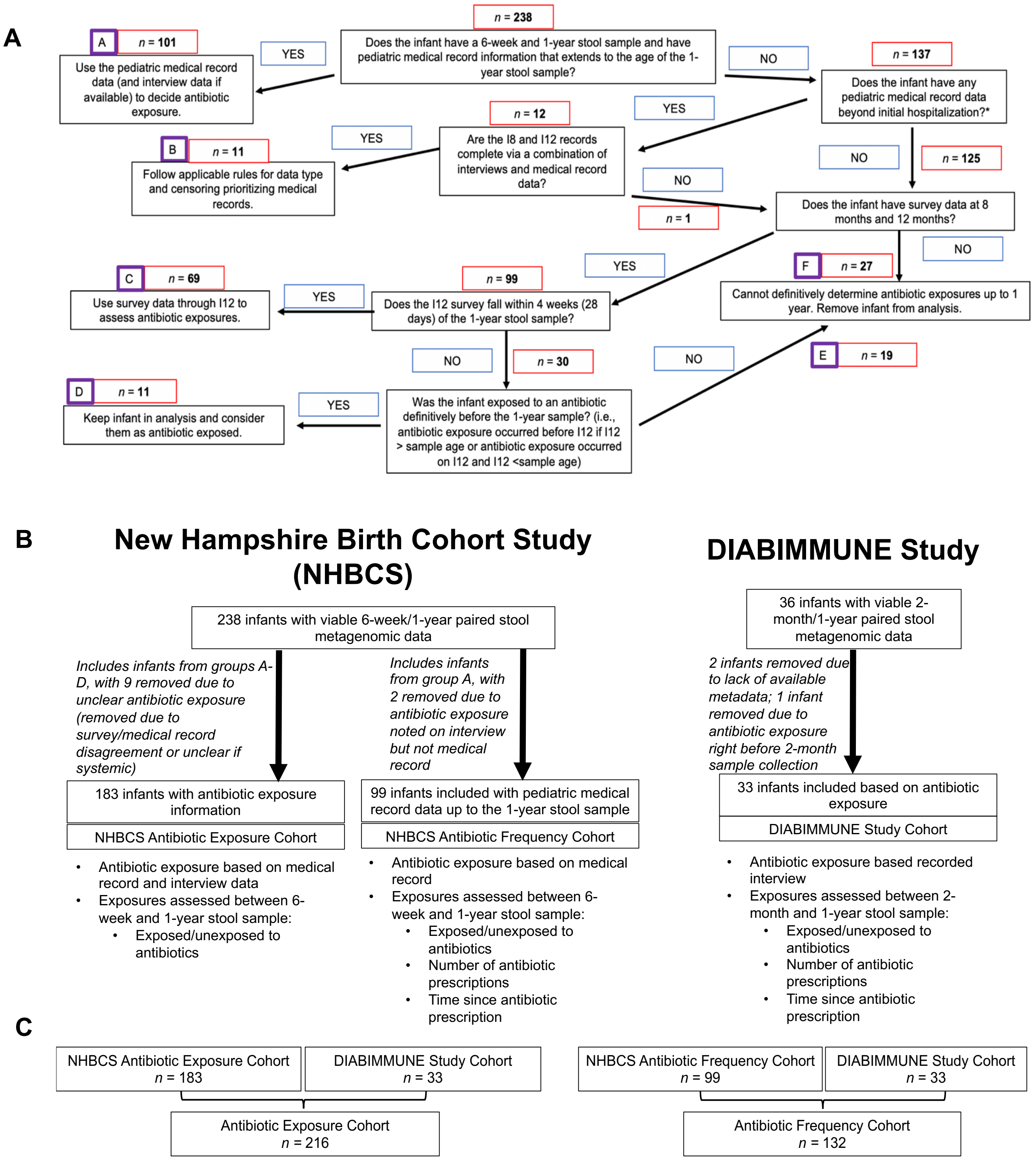

Figure S1: Description of study inclusion criteria and schema for infants. **A**) Flow diagram depicting how infants could be included based on antibiotic exposure categories. *Medical records were available during the immediate post-delivery period for all infants. **B**) Depiction of sample size and additional exclusion criteria in both the New Hampshire Birth Cohort and DIABIMMUNE studies. **C**) Combined cohort sample sizes. I8 = 8-month interview, I12 = 12-month interview.

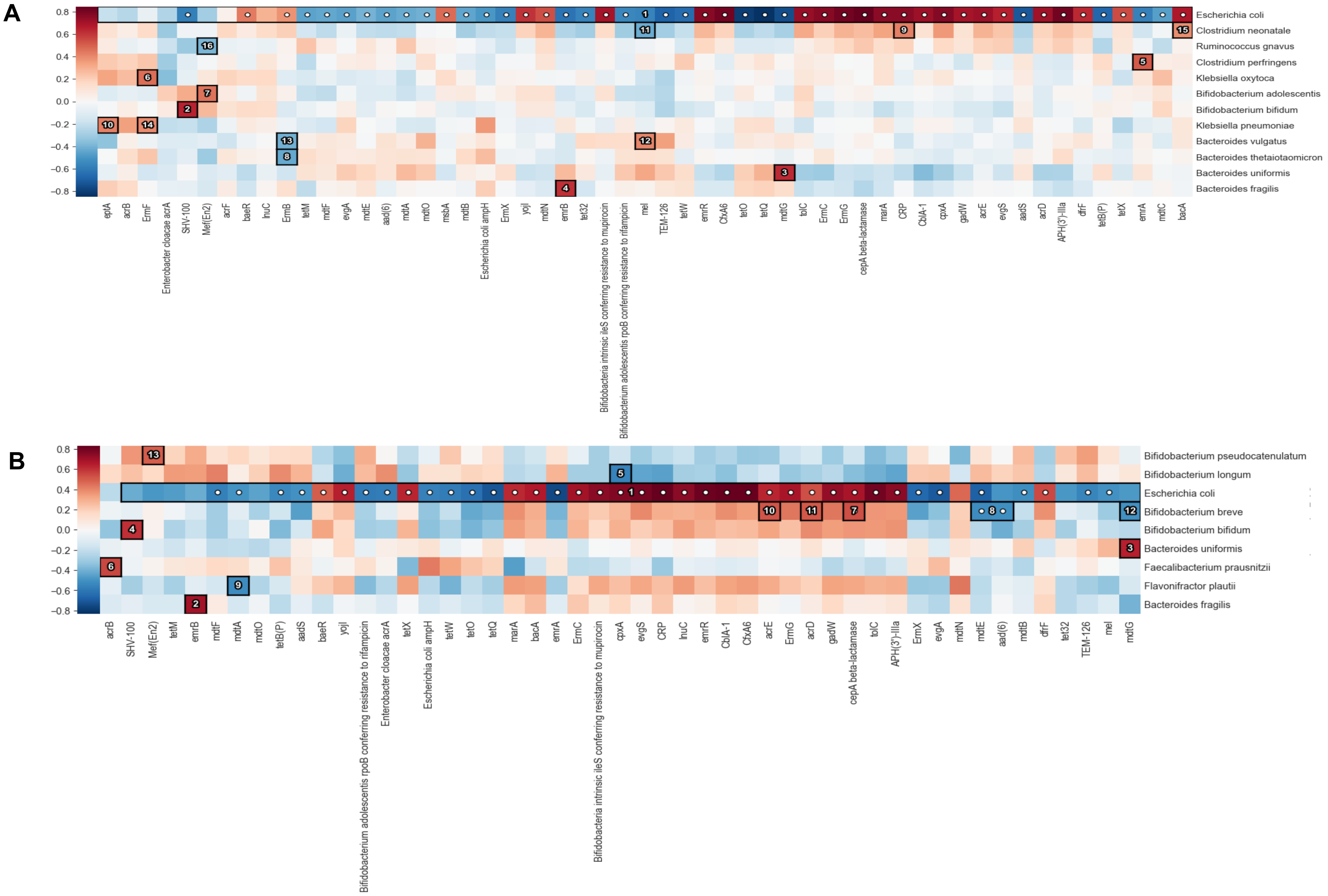
A
0.8
0.6
0.4
0.2
0.0
-0.2
-0.4
-0.6
-0.8
Escherichia coli
Clostridium neonatale
Ruminococcus gnavus
Clostridium perfringens
Klebsiella oxytoca
Bifidobacterium adolescentis
Bifidobacterium bifidum
Klebsiella pneumoniae
Bacteroides vulgatus
Bacteroides thetaiotaomicron
Bacteroides uniformis
Bacteroides fragilis
eptA
acrB
ErmF
Enterobacter cloacae acrA
SHV-100
Mef(En2)
acrF
baeR
lnuC
ErmB
tetM
mdtF
evgA
mdtE
aad(6)
mdtA
mdtO
msbA
mdtB
Escherichia coli ampH
ErmX
yojI
mdtN
emrB
tet32
Bifidobacteria intrinsic ileS conferring resistance to mupirocin
Bifidobacterium adolescentis rpoB conferring resistance to rifampicin
mel
TEM-126
tetW
emrR
CfxA6
tetO
tetQ
mdtG
tolC
ErmC
ErmG
cepA beta-lactamase
marA
CRP
CblA-1
cpxA
gadW
acrE
evgS
aadS
acrD
APH(3')-IIIa
dfrF
tetB(P)
tetX
emrA
mdtC
bacA
B
Bifidobacterium pseudocatenulatum
Bifidobacterium longum
Escherichia coli
Bifidobacterium breve
Bifidobacterium bifidum
Bacteroides uniformis
Faecalibacterium prausnitzii
Flavonifractor plautii
Bacteroides fragilis
acrB
SHV-100
Mef(En2)
tetM
emrB
mdtF
mdtA
mdtO
tetB(P)
aadS
baeR
yojI
Bifidobacterium adolescentis rpoB conferring resistance to rifampicin
Enterobacter cloacae acrA
tetX
Escherichia coli ampH
tetW
tetO
tetQ
marA
bacA
emrA
ErmC
Bifidobacteria intrinsic ileS conferring resistance to mupirocin
cpxA
evgS
CRP
lnuC
emrR
CblA-1
CfxA6
acrE
ErmG
acrD
gadW
cepA beta-lactamase
tolC
APH(3')-IIIa
ErmX
evgA
mdtN
mdtE
aad(6)
mdtB
dfrF
tet32
TEM-126
mel
mdtG

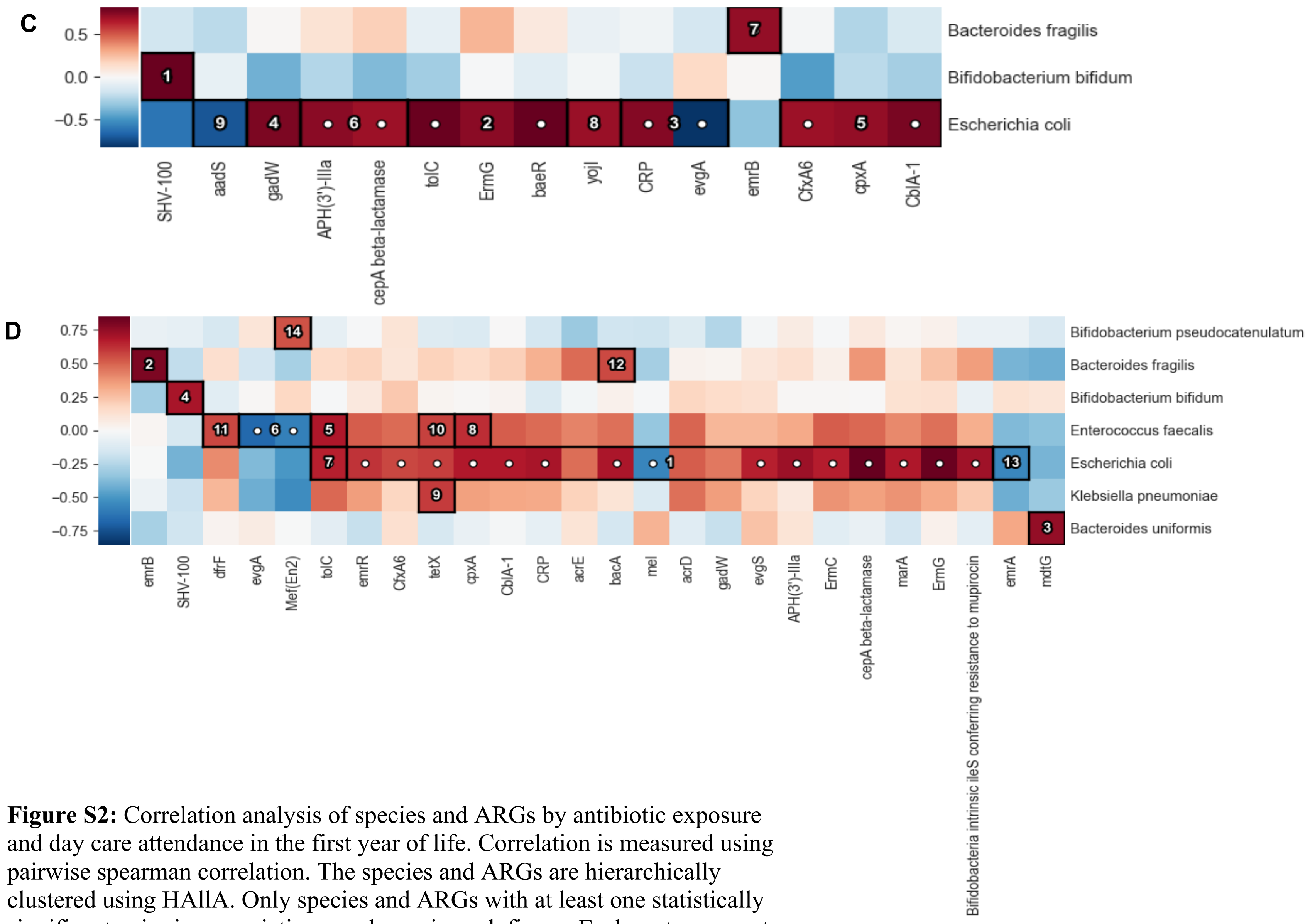

Figure S2: Correlation analysis of species and ARGs by antibiotic exposure and day care attendance in the first year of life. Correlation is measured using pairwise spearman correlation. The species and ARGs are hierarchically clustered using HAllA. Only species and ARGs with at least one statistically significant pairwise association are shown in each figure. Each part represents the following: **A)** infants unexposed to antibiotics and unexposed to day care (n = 68), **B)** infants unexposed to antibiotics but exposed to day care (n = 40), **C)** infants exposed to antibiotics but unexposed to day care (n = 23), and **D)** infants exposed to antibiotics and day care (n = 36).

Alpha and beta diversity analysis

Methods to assess the alpha (within) and beta (between) sample diversity:

To estimate the richness and evenness of samples, Shannon alpha diversity at the species level was computed for each sample. Similar to relative abundance assessments, we were interested in estimating the difference in the change in Shannon index that would have occurred over the follow-up period if an infant had not been exposed to antibiotics. We used a linear mixed-effects model implemented with the *lmer()* function in the R package lme4[1]:

Shannon index ~ (intercept) + sample age (in days) + antibiotic exposure + antibiotic exposure × sampling interval (baseline vs 1 year) + covariates + (1 | infant)

Where *Shannon index* is the alpha diversity of each sample and the remaining variables are the same as the off-target microbe and ARG abundance calculation.

We analyzed the between sample or beta diversity of bacterial species in infant stool samples in the study. We used a centered-log ratio transformation and calculated the distance between samples via the Euclidean distance. This is akin to the Aitchison distance and can outperform common metrics such as the Bray-Curtis dissimilarity metric for compositional data and is a true linear difference[2,3]. Differences between samples were visualized using the first 2 components of the principal components analysis. Similar to [4], we were interested in how microbiome composition varied by the interaction between an exposure and age. Specifically, we were interested in understanding how compositional variation with respect to antibiotic exposure is affected by compositional variation by age. Dispersion of samples by covariates were analyzed for statistical significance using PERMANOVA models with the *adonis2()* function in the vegan[5] package. 1000 permutations were run for each model. Samples were removed prior to running the PERMANOVA if they had a missing value for breastfeeding duration.

Results: within (alpha) and between (beta) sample diversity of off target microbes:

We evaluated the change to the Shannon index of off-target microbes among infants who had ever received an antibiotic, an antibiotic to treat otitis media, or 2 or more courses of antibiotic between baseline and 1-year measurements. Interestingly, at baseline, infants ultimately exposed to antibiotics had a slightly greater alpha diversity than unexposed infants, but by 1 year had an overall decreased alpha diversity. After accounting for increasing compositional diversity with time, at the one year mark, antibiotic-exposed infants in the Antibiotic Exposure Cohort ($n = 216$) had on average a 0.24 (95% CI: -0.44, -0.05) decrease in alpha diversity more than they would have had they not been exposed across the two time points (**see Figure below**). This estimate was consistent across adjusted linear mixed-effects models that considered any antibiotic exposure or an antibiotic exposure specifically for otitis media (**see Tables below**) Likewise, the trend was consistent in analyses restricted to NHBCS infants and subsequently stratified by day care attendance at 1 year (**see Tables below**). We also detected a significant dose-dependent association; infants exposed to 2 or more antibiotics between baseline and one-year measurements had on average a decrease in Shannon alpha diversity of 0.34 (95% CI: -0.63, -0.04) more than they would have had they not been exposed.

Results from between sample (beta) diversity analyses of microbiome composition did not visually reveal any clear separation based on antibiotic exposure (**see Figure below**), but as predicted, showed the strong impact of sample age on between sample dispersion (**see Figure below**). While visual results did not depict a strong impact of antibiotic exposures, variation based on the interaction between antibiotic exposure and age were consistently significant across models assessing any antibiotic exposure (**see Tables below**, p-value < 0.01). Among 216 infants, the interaction between sample age and antibiotic exposure by 1 year described 2.6% of

the variation. However, the interaction for exposure to 2 or more antibiotics and age among 132 infants was neither strong nor statistically significant ($R^2 = 0.003$, *p*-value = 0.22).

Difference-in-difference models assessing antibiotic exposure and time on Shannon alpha diversity

	**Dependent variable: Shannon alpha diversity (95% CI)**		
	**Any antibiotic exposure**	**Antibiotic exposure for otitis media**	**2 or more antibiotic exposures**
Exposed to any antibiotic	0.158**		
	(0.022, 0.294)		
DID estimate for any antibiotic	-0.259†		
	(-0.446, -0.072)		
Exposed to antibiotic for OM		0.122*	
		(-0.016, 0.260)	
DID estimate for antibiotic for OM		-0.247**	
		(-0.437, -0.056)	
Other antibiotic		0.435†	
		(0.122, 0.748)	
Exposed to 2 or more antibiotics			0.109
			(-0.105, 0.322)
DID estimate for exposure 2 or more antibiotics			-0.335**
			(-0.629, -0.042)
Sample age (in days)	0.004***	0.004***	0.004***
	(0.003, 0.004)	(0.003, 0.004)	(0.003, 0.004)
Duration of breastfeeding (in days)	-0.0001	-0.0001	-0.00002
	(-0.001, 0.0004)	(-0.001, 0.0004)	(-0.001, 0.001)
C-section	-0.085	-0.105*	-0.053
	(-0.197, 0.027)	(-0.216, 0.006)	(-0.190, 0.083)
Male	0.064	0.065	0.133**
	(-0.034, 0.161)	(-0.031, 0.162)	(0.013, 0.254)
Gestational age (in weeks)	0.003	0.002	-0.01
	(-0.028, 0.033)	(-0.029, 0.032)	(-0.052, 0.032)
Enrolled in the New Hampshire Birth Cohort Study	0.162**	0.151**	0.156**
	(0.028, 0.296)	(0.019, 0.283)	(0.014, 0.297)
Exposed to neonate antibiotic	-0.132	-0.118	-0.144
	(-0.406, 0.141)	(-0.387, 0.152)	(-0.524, 0.236)

Neonate antibiotic information missing	0.1	0.099	-0.019
	(-0.317, 0.517)	(-0.311, 0.510)	(-0.812, 0.774)
Intercept	0.825	0.88	1.318
	(-0.408, 2.057)	(-0.335, 2.096)	(-0.355, 2.991)
Observations	431	431	263
Log Likelihood	-327.125	-324.944	-202.011
Akaike Information Criterion	680.251	677.888	430.023
Bayesian Information Criterion	733.11	734.813	476.461

[a]One sample was removed from analyses due to missing duration of breastfeeding
*p-value < 0.1, **p-value < 0.05, †p-value < 0.01
DID = difference-in-difference estimate
OM = otitis media

Difference-in-difference models assessing antibiotic exposure and time on Shannon alpha diversity in NHBCS infants

	Dependent variable: Shannon alpha diversity (95% CI)		
	**NHBCS**	**No day care**	**[b]Attend day care**
Exposed to any antibiotic	0.118	0.037	0.213**
	(-0.028, 0.264)	(-0.200, 0.274)	(0.007, 0.418)
DID estimate for any antibiotic	-0.207**	-0.187	-0.362**
	(-0.411, -0.003)	(-0.503, 0.129)	(-0.648, -0.075)
Sample age (in days)	0.004***	0.004***	0.004***
	(0.003, 0.004)	(0.003, 0.004)	(0.003, 0.005)
Duration of breastfeeding (in days)	-0.0001	-0.0002	0.00002
	(-0.001, 0.0005)	(-0.001, 0.001)	(-0.001, 0.001)
C-section	-0.082	-0.055	-0.117
	(-0.195, 0.031)	(-0.224, 0.114)	(-0.330, 0.095)
Male	0.022	0.07	-0.092
	(-0.081, 0.125)	(-0.094, 0.234)	(-0.254, 0.070)
Gestational age (in weeks)	0.009	-0.001	0.014
	(-0.023, 0.041)	(-0.047, 0.045)	(-0.043, 0.072)
Exposed to neonate antibiotic	-0.084	-0.135	-0.038
	(-0.370, 0.202)	(-0.665, 0.395)	(-0.439, 0.364)
Neonate antibiotic information missing	0.131	0.053	
	(-0.274, 0.536)	(-0.398, 0.504)	
Intercept	0.759	1.191	0.564
	(-0.507, 2.025)	(-0.617, 2.999)	(-1.753, 2.880)
Observations[a]	365	182	151
Log Likelihood	-273.18	-150.926	-115.934
Akaike Inf. Crit.	570.36	325.853	253.867
Bayesian Inf. Crit.	617.159	364.301	287.057

*p-value < 0.1, **p-value < 0.05, †p-value < 0.01

NHBCS = New Hampshire Birth Cohort Study

DID = difference-in-difference estimate

[a]One sample was removed from analyses due to missing duration of breastfeeding. 32 infants from NHBCS did not have day care attendance information available.

[b]The model specified with a random effect for each infant had a singular fit so the results are equivalent to a simple linear regression model (i.e., with no random effects).

PERMANOVA analyses to assess between sample variation

	Degrees of freedom	Sum of Squares	R^2	F	Pr(>F)
**PERMANOVA for any antibiotic exposure (not adjusted for covariates)**					
Age:antibiotic exposed	1	26246.31	0.026182	12.68681	0.000999
Antibiotic exposed	1	13780.44	0.013746	6.66112	0.000999
1-year samples	1	77006.47	0.076816	37.22299	0.000999
Residual	428	885441.1	0.883256		
Total	431	1002474	1		
**PERMANOVA for any antibiotic exposure (adjusted for covariates)**					
Age:antibiotic exposed	1	26226.23	0.026195	14.52555	0.000999
Antibiotic exposed	1	13806.88	0.013791	7.647017	0.000999
Sample age (in days)	1	78250.47	0.078158	43.33947	0.000999
Duration of breastfeeding (in days)	1	8117.569	0.008108	4.495962	0.000999
C-section	1	6831.47	0.006823	3.783649	0.000999
Male	1	2212.397	0.00221	1.225349	0.113886
Gestational age (in weeks)	1	2384.025	0.002381	1.320406	0.091908
Enrolled in NHBCS	1	13555.7	0.01354	7.507901	0.000999
Exposed to neonatal antibiotics	2	4247.437	0.004242	1.176234	0.153846
Subject	208	462781	0.462234	1.232278	0.000999
Residual	212	382771.2	0.382318		
Total	430	1001184	1		
**PERMANOVA for antibiotic exposure for otitis media (OM)**					
Age:antibiotic exposed OM	1	22868.41	0.022841	12.66103	0.000999
Antibiotic exposed OM	1	14409.83	0.014393	7.977965	0.000999
Other antibiotic exposure between baseline and 1 year	1	3485.617	0.003481	1.929802	0.010989
Sample age (in days)	1	80874.23	0.080779	44.7758	0.000999
Duration of breastfeeding (in days)	1	7959.097	0.00795	4.406533	0.000999
C-section	1	7067.072	0.007059	3.912666	0.000999
Male	1	2226.315	0.002224	1.232593	0.114885
Gestational age (in weeks)	1	2415.789	0.002413	1.337495	0.093906
Enrolled in NHBCS	1	13356.58	0.013341	7.394833	0.000999

Exposed to neonatal antibiotics	2	4179.95	0.004175	1.157109	0.158841
Subject	207	459426.2	0.458883	1.228793	0.000999
Residual	212	382915.3	0.382462		
Total	430	1001184	1		
**PERMANOVA for any antibiotic exposure in NHBCS infants**					
Age:antibiotic exposed	1	25977.23	0.030001	14.0217	0.000999
Antibiotic exposed	1	13763.03	0.015895	7.428857	0.000999
Sample age (in days)	1	72899.78	0.084191	39.34903	0.000999
Duration of breastfeeding (in days)	1	7464.572	0.008621	4.029143	0.000999
C-section	1	5300.737	0.006122	2.861172	0.000999
Male	1	2212.328	0.002555	1.194146	0.152847
Gestational age (in weeks)	1	2075.299	0.002397	1.120181	0.215784
Exposed to neonatal antibiotics	2	4563.442	0.00527	1.231602	0.094905
Subject	176	400008.5	0.461963	1.226773	0.000999
Residual	179	331623.5	0.382986		
Total	364	865888.4	1		
**PERMANOVA for any antibiotic exposure –no day care attendance**					
Age:antibiotic exposed	1	9350.218	0.021675	5.03773	0.000999
Antibiotic exposed	1	4879.059	0.01131	2.62875	0.000999
Sample age (in days)	1	38599.25	0.089478	20.79658	0.000999
Duration of breastfeeding (in days)	1	6749.049	0.015645	3.636267	0.000999
C-section	1	4353.797	0.010093	2.345748	0.001998
Male	1	2149.57	0.004983	1.15815	0.183816
Gestational age (in weeks)	1	2405.698	0.005577	1.296147	0.084915
Exposed to neonatal antibiotics	2	3751.358	0.008696	1.010582	0.397602
Subject	84	195814.1	0.453921	1.255966	0.000999
Residual	88	163331.3	0.378622		
Total	181	431383.5	1		
**PERMANOVA for any antibiotic exposure –attended day care**					
Age:antibiotic exposed	1	15175.5	0.048271	9.509171	0.000999
Antibiotic exposed	1	8080.576	0.025703	5.063396	0.000999
Sample age (in days)	1	28523.3	0.090728	17.87308	0.000999

Duration of breastfeeding (in days)	1	3968.684	0.012624	2.48683	0.001998
C-section	1	2541.245	0.008083	1.592378	0.042957
Male	1	1799.857	0.005725	1.127815	0.233766
Gestational age (in weeks)	1	2021.527	0.00643	1.266716	0.132867
Exposed to neonatal antibiotics	1	1881.218	0.005984	1.178796	0.178821
Subject	70	135488.3	0.430965	1.212839	0.000999
Residual	72	114903.4	0.365488		
Total	150	314383.6	1		
**PERMANOVA for exposure to 2 or more antibiotics**					
Sample age (in days)	1	66065.96	0.108962	37.0134	0.000999
Exposed 2+ antibiotics	1	2929.96	0.004832	1.641507	0.01998
Age:exposed 2+ antibiotics	1	1982.269	0.003269	1.110565	0.228771
Duration of breastfeeding (in days)	1	5783.613	0.009539	3.240264	0.000999
C-section	1	5607.135	0.009248	3.141393	0.000999
Male	1	2339.099	0.003858	1.310478	0.097902
Gestational age (in weeks)	1	2341.366	0.003862	1.311749	0.108891
Enrolled in NHBCS	1	11405.5	0.018811	6.389921	0.000999
Exposed to neonatal antibiotics	2	4756.89	0.007846	1.332522	0.046953
Subject	124	274637.1	0.452958	1.240848	0.000999
Residual	128	228469.8	0.376815		
Total	262	606318.6	1		

: used to indicate an interaction term

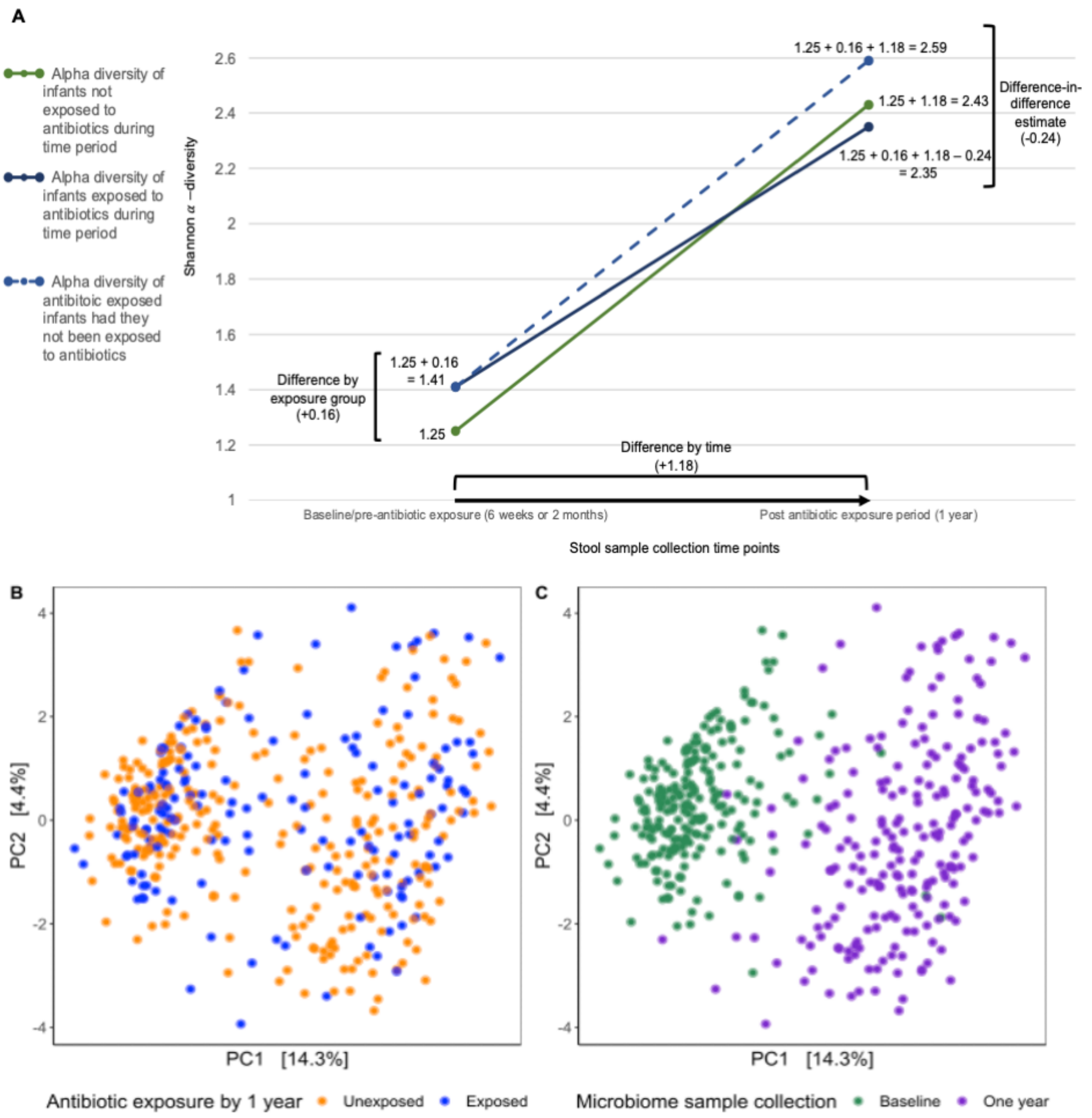

Figure: Within and between sample species diversity by antibiotic exposure and time period in 216 infants. **A**) A conceptual model depicting the average within sample (alpha diversity) difference by antibiotic exposure (yes/no) and time period (pre/post antibiotic exposure). Estimates are derived from a crude linear regression model with the Shannon index per sample (n= 432) as the outcome and antibiotic exposure ever, baseline/1-year treatment, and the difference-in-difference estimate for the interaction as independent variables. All differences are statistically significant at $\alpha = 0.05$. The solid lines represent the trajectory of the Shannon index by group observed in this study. The lighter blue dashed line depicts the alpha diversity in a counterfactual scenario in which antibiotic-exposed infants were never exposed to antibiotics. **B** and **C**) Principal component analysis of between sample diversity. The relative abundance of species was centered log-transformed and plotted using the Euclidean distance. Samples are colored by **B** antibiotic exposure by 1 year (excluding antibiotic exposure immediately following birth) or **C** age of the infant at sample collection.

Discussion: Antibiotics' impacts on microbial diversity

Previous studies have found mixed results associating diversity and antibiotic exposure depending on geography, age of the child, type of antibiotic prescribed, and time since antibiotic exposure, but, generally, antibiotic exposures are associated with stable or decreased within-sample diversity[6]. Consistently, we found NHBCS infants had a statistically significantly (p-value < 0.05) higher alpha diversity than samples in the DIABIMMUNE Study. This may be due to true differences in alpha diversity between the cohorts or lower sequencing depths for samples in the DIABIMMUNE Study. In the DIABIMMUNE cohort study[7], on average, infants without antibiotic exposures had a microbiome with higher richness, but this was only evident after the first year of life. In our study, we consistently found that there was an inverse association between antibiotic exposure and Shannon alpha diversity prior to 1 year. Interestingly, however, at the baseline timepoint (at approximately 6 weeks of life) infants who were ultimately exposed to antibiotics had higher alpha diversity than infants not exposed to antibiotics. This emphasizes the importance of considering timing of exposure, in particular pre- and post-antibiotic exposure measurement, to discern trends over time.

Antibiotic exposure classification and sensitivity analyses – methods

Infants in the NHBCS have a range of questionnaire and pediatric medical record data available over the course of their first year of life. Pediatric medical record data is derived from medical record review from a trained professional and includes detailed antibiotic information on the timing, type, and reason for antibiotic prescriptions. Antibiotic exposure is assessed on interviews occurring at approximately 4, 8, and 12 months of life and asks caregivers to identify the type and reason that an antibiotic was given to a child. Through assessment of infants within the NHBCS, we identified that infants with paired 6-week/1-year stool samples had various completion levels for both pediatric medical record and interview data through the first year of life. We classified antibiotic exposure in two ways in an attempt to maximize sample size and minimize exposure misclassification. Thus, we created one cohort that would be used to assess antibiotic exposure vs. no exposure (NHBCS Antibiotic Exposure Cohort) and the other that would assess type and number of antibiotic prescriptions (NHBCS Antibiotic Frequency Cohort). Sample sizes including a flow diagram (**Figure S1**) and results from sensitivity analyses are available below.

NHBCS Antibiotic Exposure Cohort:

The conceptual exposure for this cohort was antibiotics given to infants between the baseline (6-week) and 1-year stool sample collection that could impact the gut (i.e., ingested or administered systemically).

Operationally, the antibiotic exposures cohort required more protocols to ensure that antibiotic exposures classified via pediatric medical records and interview data were captured correctly.

Depending upon the data available, these were the protocols used to assign infants' antibiotic exposures:

- If the infant had both pediatric medical record and interview data available, only include infants that had concordant pediatric medical record and interview data indicating an antibiotic exposure.
- If the infant had only interview or pediatric medical record data but that indicated an antibiotic exposure during the time period of interest, mark them as antibiotic exposed.
- If the infant had only interview or pediatric medical record data but that indicated no antibiotic exposure during the time period of interest, mark them as antibiotic unexposed.

Determining if the antibiotic exposure occurred before or after the stool sample was also considered. With pediatric medical record data, the time (days since birth) of the antibiotic prescription was given providing antibiotic exposure timing. However, antibiotic exposures as measured on the interview at 12 months could occur slightly before or after the stool sample was collected. Based on these challenges, we created a schema to determine how to include infants based only on interview data or pediatric medical record data. Since prescription dates are given for pediatric medical record data, the time of antibiotic prescriptions are pulled from there. With interview data, one of the following conditions needed to be met in order to include an infant:

1) 12-month interview data collected within a set time interval before or after the stool sample was collected.

2) 12-month interview occurred significantly earlier than the 1-year stool sample but there was definitive evidence of an antibiotic exposure prior to the stool sample collection.
3) The 12-month interview occurred significantly after the stool sample was collected but no antibiotic exposures were noted.

The next step was determining the time interval to determine correct classification of samples. To determine the best time interval, we utilized infants that had 8- and 12-month interview data as well as pediatric medical record data beyond their first year of life as a sensitivity cohort. We assessed a 14, 21, and 28-day interval around the stool sample collection time point noting concordance and Kappa.

Finally, we applied the chosen time interval and conditions to the full dataset of infants. Based on the sensitivity analysis, the following assumptions were made:

1) 28 days (inclusive) on either side of the stool sample collection point was considered the buffer of inclusion for full records for interview data.
2) The pediatric medical record is considered the "gold standard" because it includes more detailed information on the exact date of the antibiotic prescription which cannot be assumed by the interview. Additionally, parents are less likely to be as knowledgeable about specific antibiotic prescriptions given compared to clinicians.
3) Infants prescribed an antibiotic on the pediatric medical record were actually given an antibiotic.
4) If interview data mentions an antibiotic was given but it's not matched on the pediatric medical record, it was considered an antibiotic exposure, but ultimately that infant would

be censored as there is disagreement between the medical record and interview (i.e., the assumption is that no antibiotic prescriptions on the medical records were missed among infants with full medical records).

5) Infants noted as receiving an antibiotic on the interview data, were given the antibiotic.
6) On the interview, we assume recall bias is minimal as parents would likely know if an infant is given an antibiotic for a condition or not (i.e., there's a high likelihood that parents would remember either way). There was concern about recall bias for specificity of conditions. In other words, on a medical record the clinician would often say an antibiotic was for "otitis media" while on the interview it may list a more general list of symptoms for why the antibiotic was given (e.g., ear infection, runny nose, and fever). While this doesn't change the fact that the infant was exposed to an antibiotic, it does limit the ability to compare between the two classification systems.
7) Infants only needed a completed interview at 8 and 12 months as the vast majority of antibiotic exposures occurred after approximately 6 months.
8) Infants that had an antibiotic exposure noted on the 4-month interview were exposed to an antibiotic after the baseline sample was collected. This assumption was based on the sensitivity cohort in which no infant with pediatric medical record data had an antibiotic exposure before 6 weeks unless they were initially given one at birth which is noted in the infant medical record.

NHBCS Antibiotic Frequency Cohort:

The conceptual exposure in this cohort was based on the number of antibiotic prescriptions. This was operationalized by assessing all antibiotic prescription from all infants with pediatric medical records up to the 1-year stool sample collection.

Ultimately, the decision was made to include infants that have a recorded antibiotic prescription even if it was not noted on the interview data. We made this assumption because we do not know in infants with missing interview data if 1) the antibiotic prescription would have been captured, 2) antibiotic prescriptions are missing from interview data due to the infant not redeeming their antibiotic prescription, or 3) the caregiver did not recall an antibiotic exposure for his/her/their child.

Antibiotic exposure classification and sensitivity analyses – results

NHBCS Antibiotic Exposure Cohort:

To assess the time interval that could be used to count antibiotic exposures among infants with interview data in NHBCS Antibiotic Exposure Cohort, we assessed concordance between interview and medical record data at 14, 21, and 28 day intervals among infants with both interview and pediatric medical record data available. 81 infants had pediatric medical record data at least 28 days beyond when their 1-year stool sample was collected as well as having an 8-month and 12-month interview available.

		**Pediatric medical record**	
		**Exposed to antibiotic**	**Unexposed to antibiotic**

**Interview occurred within 28 days of stool sample or infant could be classified based on other condition***	**Exposed to antibiotic**	22	1
	**Unexposed to antibiotic**	2	43

Concordance: 65/68 = 95.6%, Kappa = 90.2%

		**Pediatric medical record**	
		**Exposed to antibiotic**	**Unexposed to antibiotic**
**Interview occurred within 21 days of stool sample or infant could be classified based on other condition***	**Exposed to antibiotic**	22	0
	**Unexposed to antibiotic**	2	32

Concordance: 54/56 = 96.4%, Kappa = 92.6%

		**Pediatric medical record**	
		**Exposed to antibiotic**	**Unexposed to antibiotic**
**Interview occurred within 14 days of stool sample or infant could be classified based on other condition***	**Exposed to antibiotic**	22	0
	**Unexposed to antibiotic**	2	28

Concordance: 50/52 = 96.2%, Kappa = 92.2%

*Other conditions included: 12-month interview occurred significantly earlier than the 1-year stool sample but there was definitive evidence of an antibiotic exposure prior to the stool sample collection. The 12-month interview occurred significantly after the stool sample was collected but no antibiotic exposures were noted.

Using the sensitivity cohort for the 28-day interval (i.e., the cohort with full pediatric medical record and interview data above), we would have included 65 infants (out of the 68, the 3

incorrect classifications would have been censored). Of those 65 infants, 22 were exposed to an antibiotic. This provides an incidence of antibiotic exposure in the cohort of 22/65 or 33.8%. For any antibiotic exposure for otitis media [not shown], the concordance is still high. In the sensitivity set (n = 68), 4 infants were censored based on unclear antibiotic for otitis media exposure. This left 64 infants that were correctly able to be classified and 19 were exposed to antibiotics for otitis media among them (incidence = 19/64 or 29.7%). Lastly, in the 28-day sensitivity set (n = 68) and censoring for unclear antibiotic exposure for any reason or for unclear otitis media diagnosis, 5 infants would have been censored for antibiotic exposure in general or for antibiotic exposure for otitis media. 20 infants were exposed to antibiotics in general and 19 for otitis media. This makes the incidence 20/63 and 19/63 or 31.7% and 30.2% respectively.

The final NHBCS Antibiotic Exposure Cohort only included infants that were not censored for unclear antibiotic exposure or unclear antibiotic exposure for otitis media ultimately consisting of 183 infants. Of which, 63 were exposed to any antibiotic (incidence = 34.4%), and 58 to an antibiotic for otitis media (incidence = 31.7%).

Thus, our sensitivity analysis showed strong concordance and agreement between the incidence of antibiotic exposures in the sensitivity and ultimate NHBCS Antibiotic Exposure Cohort.

NHBCS Antibiotic Frequency Cohort:

Similarly to the sensitivity analysis for the NHBCS Antibiotic Exposure Cohort, we utilized a sensitivity cohort to assess agreement between interview and antibiotic prescriptions data per infant. We included infants in this cohort based on slightly different metrics than the NHBCS

Antibiotic Exposure Cohort. Infants were included if they had interview data at 8 and 12 months with the 12-month interview occurring within 28 days of the stool sample along with full pediatric medical record data (n = 64). Additionally, 2 infants were included because they had interview and medical record data beyond the point of their sample collection with no antibiotic exposure. In this sensitivity analysis cohort of 66 infants we found the following results:

		Pediatric medical record	
		Antibiotic(s) noted	Antibiotic(s) not noted
Interview	Antibiotic(s) noted	13	1
	Antibiotic(s) not noted	7	45

Concordance = (13+45)/66 = 87.8%, Kappa = 68.6%

Note: If multiple antibiotic prescriptions were noted, agreement was only classified if all antibiotic prescriptions were captured in both.

This table demonstrates that there is substantial agreement between methods; however, as expected, some of the antibiotic prescriptions are not captured on the interview. This could be due to a lack of reporting of the antibiotic exposure or that the antibiotic was not given to the infant even when it was prescribed. Given this uncertainty, we decided to include all antibiotic prescriptions. Based on this sensitivity set, if bias was present, we believe this would bias our results towards the null.

Covariates

Extensive data on lifestyle, medical history, and environmental exposures were collected from participants in the NHBCS; select covariate data were available for the DIABIMMUNE Study. Across both cohorts, covariates were selected as a result of an *a priori* literature review and a theorized association with antibiotic exposure and the infant gut microbiome. We included gestational age (in weeks)[8], duration of breastfeeding (in days)[9–11], delivery mode (vaginal or cesarean)[11–13], sex (male or female)[14], infant age at stool sample collection (in days)[12], and antibiotic usage immediately following birth (yes/no)[15]. Infants were excluded if they had a known antibiotic exposure after the period immediately following birth but before the baseline microbiome sample was collected. Additionally, for NHBCS data, we considered day care attendance by 1 year (yes/no) as a covariate as it had previously been associated with otitis media[16,17] and we hypothesized that it could have an association with the gut microbiome[18,19]. Adjusted models including infants from both the NHBCS and DIABIMMUNE Study also included a dichotomous covariate to adjust for study origin.

Stool microbiome assessment

NHBCS samples:

Stool collection and metagenomic DNA shotgun sequencing have been previously completed and described for our cohort[20,21]. In brief, infant stool samples were collected in diapers at approximately 6 weeks and 1 year of age. DNA was extracted from the stool samples with concentrations of DNA samples used for sequencing ranging from 1 ng/ul to 25 ng/ul. Sequencing was conducted at the Marine Biological Laboratory in Woods Hole, MA using

established protocols. A Covaris S220 focused ultrasonicator sheared DNA samples to a mean size of 400 base pairs and libraries were constructed using Nugen's Ovation Ultralow V2 protocol.

All samples were processed using KneadData as part of the bioBakery3 pipeline[22]. This pipeline included samples that were sequenced and merged as paired end reads or samples that were only sequenced as forward reads. Previously, we identified that using both single and paired end reads did not affect associations with early-life factors[20]. Infants were excluded if they did not have both a 6-week and 1-year stool sample. This left 238 infants and 476 total samples. Average sequencing depth for paired (n = 237) and single-read (n = 239) samples were 61.1 (standard deviation of 21.9) and 24.5 (standard deviation of 10.5) millions of reads respectively.

DIABIMMUNE Study samples:

Information regarding stool sample collection and quality control procedures are outlined in the primary research papers by the DIABIMMUNE Study group[7,23]. Stool metagenomic datasets used for this analysis were downloaded directly from the DIABIMMUNE Study website's Antibiotics Cohort metagenomic forward read sequencing data[24]. Infants were excluded from this analysis if they did not have a 2-month baseline or 1-year sample. Average sequencing depth of the remaining samples (n = 66) was 10.6 million reads with a standard deviation of 7.6 million reads.

Taxonomic and antibiotic resistance gene profiling:

For samples from both the NHBCS and DIABIMMUNE Studies, taxonomy down to the species level was assigned using MetaPhlAn3[25] . Only bacterial taxa were considered. Taxa that were identified in less than 1% of samples (assessed in the Antibiotic Exposure and Frequency Cohorts) were excluded in further analyses. After prevalence filtering, the relative abundance at each level of interest (phyla, genera, and species) were normalized to sum to 1.

ARGs were profiled using ShortBRED[26]. ShortBRED works in two steps. It first creates a set of representative ARG markers from a database of ARG sequences. It then uses this set of highly sensitive and specific markers to probe for sequences in the shotgun metagenomic data that likely confer antibiotic resistance. ARGs of interest were derived from the Comprehensive Antibiotic Resistance Database (CARD)[27] v3.1.1 antibiotic resistance gene ontologies version dated January 29, 2021 using sequences derived from CARD's protein FASTA protein homolog model. These proteins of interest were mapped using the shortbred_identify.py script with UniRef90[28] as the reference protein database[29]. Resulting markers for ARGs were then quantified in the NHBCS sequence datasets using the shortbred_quantify.py script with default settings. Outputs from ShortBRED are normalized for average read length, marker length, and sequencing depth and are represented in reads per kilobase of reference sequence per million samples reads (RPKM). ARGs identified in fewer than 1% of all samples in the Antibiotic Exposure and Frequency Cohorts were removed.

Statistical analyses

Assessing individual off-target microbes and antibiotic resistance genes:

Our assessment of off-target microbes and ARGs integrated the strengths of the cohort studies; specifically, longitudinal metadata, medical record data, and microbiome data. We were interested in estimating the difference in relative abundance that would have occurred over the follow-up period if an infant had not been exposed to antibiotics.

Estimation of the difference-in-difference estimate was performed using Microbiome Multivariable Associations with Linear Models (MaAsLin2)[30]. Default options were used with the exception of the following, which aided in the interpretation of our results: no transformations were made to assess microbial abundance calculations and no transformations or normalizations were used for ARG assessment. A 95% confidence interval was computed from the output for the difference-in-difference effect estimate's standard error in MaAsLin2.

Modeling antibiotic exposure on overall antibiotic resistance gene abundance:

To estimate difference-in-difference estimates for the overall ARG abundance per sample, we ran fixed-effects linear regression models with ARG abundance in RPKM as the outcome.

The general formula for these analyses was the following:

Overall ARG abundance ~ (intercept) + sample age (in days) + antibiotic exposure + antibiotic exposure × sampling interval (baseline vs 1 year) + covariates

The same covariates as the individual microbe and ARG model were used. Difference-in-difference estimates with 95% confidence intervals are reported.

Species and antibiotic resistance gene correlation analyses:

HAllA version 0.8.18 was used to identify blocks and pairs of species and ARGs that were associated with each other[31]. HAllA uses a three-step system that calculates pairwise associations between ‘omics datasets, hierarchically clusters each dataset, and then iteratively identifies associated blocks of features. We assessed the correlation based on the difference between the baseline and 1-year relative abundance of each species and ARG. Species and ARGs with greater than a 0.5% mean abundance in the Antibiotic Exposure Cohort were included. Species and ARG abundances were centered log-ratio transformed using the microbiome[32] package to account for relative abundance data. Samples were stratified by antibiotic exposure and day care attendance at 1 year before running them through HAllA. Spearman correlation with a Benjamini-Hochberg correction of 0.05 was used to associate features and evaluate statistical significance.

Model sensitivity analyses:

In addition to crude and adjusted models for abundance and diversity metrics, we estimated difference-in-difference or interaction estimates for two other antibiotic exposure scenarios. One set of sensitivity models considered antibiotic exposure specifically for otitis media with an added variable to account for antibiotic exposures not for otitis media (tested with the Antibiotic Exposure Cohort). In the other model sets, infants were only considered exposed if they received two or more antibiotic prescriptions (tested with the Antibiotic Frequency Cohort).

Based on assumptions of the difference-in-difference modeling approach, we stratified infants in the NHBCS cohort based on day care attendance. Difference-in-difference regression was used to evaluate if antibiotic exposure impacted abundance or diversity metrics differentially by the two groups.

Difference-in-differences assumptions and sensitivity analyses

Difference-in-differences assumptions:

In order for the difference-in-differences approach to represent an unbiased amount of change in the population due to an antibiotic, a few assumptions must be met[33,34].

The first assumption (often called the strict exogeneity assumption) is that the intervention cannot be associated with the outcome at baseline. While this may *not* be true if we were considering microbes targeted for treatment by the antibiotic exposures, in this case we were only interested in off-target microbes and antibiotic resistance genes.

A second assumption (often called the positivity assumption) is that all people in the study could have been given the intervention. We assume in this study that, since all infants are involved in the medical system to participate in cohort studies, that they all have access to antibiotics.

The last assumption is the parallel trends assumption. This assumption says that there are no unmeasured variables that are both time and group varying. Graphically this can be depicted using two lines to represent the relative abundance of a microbe or ARG among those exposed and unexposed prior to the intervention. If the lines are not parallel, the assumption is not met. These lines are often depicted as linear, but this is not a prerequisite. While this assumption is challenging to address with data at only 2 time points, care has been taken to evaluate variables that could be considered time and group variant. Unless otherwise mentioned, we assume that covariates are time invariant and that the effect of the covariates on our outcome at baseline is

also time invariant. This is why we did not assess the interaction between time and other covariates.

Sensitivity analyses:

In particular, we explored sample age, duration of breastfeeding, exclusive breastfeeding at the sample collection, and exposure to day care by 1 year due to time variance (Supplemental tables included below). Sample age, duration of breastfeeding, and exclusive breastfeeding at sample collection did not vary significantly by group in the Antibiotic Exposure Cohort (n = 216; Kruskal-Wallis or chi-squared p-value in baseline and one year > 0.05). However, at baseline, infants eventually exposed to an antibiotic were breastfed slightly longer than infants not exposed to an antibiotic (48.6 days vs. 44.8 days, Kruskal-Wallis p-value = 0.054). Due to this small difference in days breastfeeding and non-significant differences in exclusive breastfeeding status, we did not consider this to be a major factor affecting results and did not stratify analyses based on duration or exclusivity of breastfeeding. Since we only had exposure to daycare available in the NHBCS cohorts, we only explored this in NHBCS infants (Supplemental table included below). Among the 183 infants in the Antibiotic Exposure Cohort, infants that were given an antibiotic were also more likely to attend daycare. Thus, in order to tease apart the effects of daycare and antibiotic exposure, we conducted sensitivity analyses among infants that attended and didn't attend daycare by 1 year.

Exposure to antibiotic by 1 year in Antibiotic Exposure Cohort

	**Not exposed**	**Exposed**	**Overall**
	**N = 140**	**N = 76**	**N = 216**
**Sample age in days of baseline sample**			
Mean (SD)	48.1 (15.6)	49.8 (17.4)	48.7 (16.2)
Median [Min, Max]	44.0 [28.0, 150]	45.0 [17.0, 150]	44.0 [17.0, 150]
**Sample age in days of one year sample**			
Mean (SD)	368 (27.1)	375 (28.3)	371 (27.6)
Median [Min, Max]	368 [319, 570]	371 [325, 477]	369 [319, 570]
**Duration of breastfeeding in days at baseline**			
Mean (SD)	44.8 (19.0)	48.6 (18.6)	46.2 (18.9)
Median [Min, Max]	43.0 [0, 150]	45.0 [0, 150]	44.0 [0, 150]
Missing	1 (0.7%)	0 (0%)	1 (0.5%)
**Exclusive breastfeeding at baseline**			
No	49 (35.0%)	21 (27.6%)	70 (32.4%)
Yes	90 (64.3%)	55 (72.4%)	145 (67.1%)
Missing	1 (0.7%)	0 (0%)	1 (0.5%)
**Exclusive breastfeeding at 1 year**			
No	95 (67.9%)	56 (73.7%)	151 (69.9%)
Yes	45 (32.1%)	20 (26.3%)	65 (30.1%)
**Duration of breastfeeding in days at 1 year**			
Mean (SD)	245 (134)	265 (117)	252 (128)
Median [Min, Max]	317 [0, 449]	304 [0, 392]	306 [0, 449]
**Delivery mode**			
Vaginal	101 (72.1%)	59 (77.6%)	160 (74.1%)
C-section	39 (27.9%)	17 (22.4%)	56 (25.9%)
**Sex**			
Female	55 (39.3%)	29 (38.2%)	84 (38.9%)
Male	85 (60.7%)	47 (61.8%)	132 (61.1%)
**Gestational age (in weeks)**			
Mean (SD)	39.1 (1.66)	39.0 (1.72)	39.1 (1.68)
Median [Min, Max]	39.1 [29.1, 42.6]	39.1 [33.3, 41.9]	39.1 [29.1, 42.6]

Descriptive overview of infants in New Hampshire Birth Cohort Study Antibiotic Exposure Cohort

	Unexposed to antibiotic	Exposed to antibiotic	Overall
	**(N=120)**	**(N=63)**	**(N=183)**
**Sample age at 6-week sample (days)**			
Mean (SD)	45.7 (14.7)	46.2 (15.1)	45.8 (14.8)
Median [Min, Max]	43.0 [28.0, 150]	44.0 [17.0, 150]	43.0 [17.0, 150]
**Sample age of 1 year samples (days)**			
Mean (SD)	369 (28.5)	376 (30.2)	371 (29.2)
Median [Min, Max]	369 [320, 570]	371 [325, 477]	370 [320, 570]
**Maternal BMI (kg/m2)**			
Mean (SD)	26.1 (5.77)	26.5 (5.97)	26.2 (5.83)
Median [Min, Max]	24.4 [17.4, 48.2]	24.9 [19.0, 47.2]	24.8 [17.4, 48.2]
**Delivery method**			
Vaginal delivery	84 (70.0%)	47 (74.6%)	131 (71.6%)
C-section	36 (30.0%)	16 (25.4%)	52 (28.4%)
**Gestational age at birth (weeks)**			
Mean (SD)	39.0 (1.66)	38.9 (1.72)	38.9 (1.67)
Median [Min, Max]	39.0 [29.1, 41.1]	39.0 [33.3, 41.9]	39.0 [29.1, 41.9]
**Parity**			
0	61 (50.8%)	28 (44.4%)	89 (48.6%)
1+	54 (45.0%)	34 (54.0%)	88 (48.1%)
Missing	5 (4.2%)	1 (1.6%)	6 (3.3%)
**Attend day care by 1 year**			
No	68 (56.7%)	23 (36.5%)	91 (49.7%)
Yes	40 (33.3%)	36 (57.1%)	76 (41.5%)
Missing	12 (10.0%)	4 (6.3%)	16 (8.7%)
**Breastfeeding duration at 6 weeks (days)**			
Mean (SD)	41.9 (18.3)	45.0 (16.6)	43.0 (17.7)
Median [Min, Max]	42.0 [0, 150]	43.0 [0, 150]	43.0 [0, 150]
Missing	1 (0.8%)	0 (0%)	1 (0.5%)
**Exclusive breastfeeding at baseline**			
No	33 (27.5%)	11 (17.5%)	44 (24.0%)
Yes	86 (71.7%)	52 (82.5%)	138 (75.4%)
Missing	1 (0.8%)	0 (0%)	1 (0.05%)
**Breastfeeding duration at 1 year (days)**			
Mean (SD)	238 (139)	265 (119)	247 (133)
Median [Min, Max]	312 [0, 449]	304 [0, 391]	304 [0, 449]
**Exclusive breastfeeding at 1 year**			
No	75 (62.5%)	43 (68.3%)	118 (64.5%)
Yes	45 (37.5%)	20 (31.7%)	65 (35.5%)
**Sex**			
Female	46 (38.3%)	24 (38.1%)	70 (38.3%)
Male	74 (61.7%)	39 (61.9%)	113 (61.7%)
**Maternal education level**			
High school or equivalent	11 (9.2%)	5 (7.9%)	16 (8.7%)
Junior college, technical school, or some college	20 (16.7%)	6 (9.5%)	26 (14.2%)
College	38 (31.7%)	29 (46.0%)	67 (36.6%)
Post-graduate	49 (40.8%)	22 (34.9%)	71 (38.8%)
Missing	2 (1.7%)	1 (1.6%)	3 (1.6%)

References

1. Bates, D., Mächler, M., Bolker, B. M. & Walker, S. C. Fitting linear mixed-effects models using lme4. *J. Stat. Softw.* **67**, 1–48 (2015).
2. Gloor, G. B., Macklaim, J. M., Pawlowsky-Glahn, V. & Egozcue, J. J. Microbiome datasets are compositional: And this is not optional. *Front. Microbiol.* **8**, 1–6 (2017).
3. Aitchison, J., Barceló-Vidal, C., Martín-Fernández, J. A. & Pawlowsky-Glahn, V. Logratio analysis and compositional distance. *Math. Geol.* **32**, 271–275 (2000).
4. Ghosh, T. S., Das, M., Jeffery, I. B. & O'Toole, P. W. Adjusting for age improves identification of gut microbiome alterations in multiple diseases. *Elife* **9**, 1–29 (2020).
5. Oksanen, J. *et al.* vegan: Community Ecology Package. R package version 2.5.6. (2019).
6. McDonnell, L. *et al.* Association between antibiotics and gut microbiome dysbiosis in children: systematic review and meta-analysis. *Gut Microbes* **13**, 1–18 (2021).
7. Yassour, M. *et al.* Natural history of the infant gut microbiome and impact of antibiotic treatments on strain-level diversity and stability. *Sci Trans Med* **8**, 1173–1178 (2016).
8. Gasparrini, A. J. *et al.* Persistent metagenomic signatures of early-life hospitalization and antibiotic treatment in the infant gut microbiota and resistome. *Nat. Microbiol.* **4**, 2285–2297 (2019).
9. Vatanen, T. *et al.* The human gut microbiome in early-onset type 1 diabetes from the TEDDY study. *Nature* **562**, 589–594 (2018).
10. Rahman, S. F., Olm, M. R., Morowitz, M. J. & Banfield, J. F. Machine learning leveraging genomes from metagenomes identifies influential antibiotic resistance genes in the infant gut microbiome. *mSystems* **3**, 1–12 (2018).
11. Madan, J. C. *et al.* Association of cesarean delivery and formula supplementation with the

intestinal microbiome of 6-week-old infants. *JAMA Pediatr.* **170**, 212–219 (2016).

12. Bäckhed, F. *et al.* Dynamics and stabilization of the human gut microbiome during the first year of life. *Cell Host Microbe* **17**, 690–703 (2015).

13. Shao, Y. *et al.* Stunted microbiota and opportunistic pathogen colonization in caesarean-section birth. *Nature* **574**, 117–121 (2019).

14. Sinha, T. *et al.* Analysis of 1135 gut metagenomes identifies sex-specific resistome profiles. *Gut Microbes* **10**, 358–366 (2018).

15. Uzan-Yulzari, A. *et al.* Neonatal antibiotic exposure impairs child growth during the first six years of life by perturbing intestinal microbial colonization. *Nat. Commun.* **12**, (2021).

16. Kørvel-Hanquist, A., Koch, A., Lous, J., Olsen, S. F. & Homøe, P. Risk of childhood otitis media with focus on potentially modifiable factors: A Danish follow-up cohort study. *Int. J. Pediatr. Otorhinolaryngol.* **106**, 1–9 (2018).

17. Kaur, R., Morris, M. & Pichichero, M. E. Epidemiology of acute otitis media in the postpneumococcal conjugate vaccine era. *Pediatrics* **140**, 1–11 (2017).

18. Thompson, A. L., Monteagudo-Mera, A., Cadenas, M. B., Lampl, M. L. & Azcarate-Peril, M. A. Milk- and solid-feeding practices and daycare attendance are associated with differences in bacterial diversity, predominant communities, and metabolic and immune function of the infant gut microbiome. *Front. Cell. Infect. Microbiol.* **5**, 1–15 (2015).

19. Hermes, G. D. A., Eckermann, H. A., de Vos, W. M. & de Weerth, C. Does entry to center-based childcare affect gut microbial colonization in young infants? *Sci. Rep.* **10**, 1–13 (2020).

20. Lebeaux, R. M. *et al.* The infant gut resistome is associated with E. coli and early-life exposures. *BMC Microbiol.* **21**, 1–18 (2021).

21. Coker, M. O. *et al.* Specific class of intrapartum antibiotics relates to maturation of the infant gut microbiota: a prospective cohort study. *BJOG An Int. J. Obstet. Gynaecol.* **127**, 217–227 (2019).

22. Beghini, F. *et al.* Integrating taxonomic, functional, and strain-level profiling of diverse microbial communities with bioBakery 3. *Elife* **10**, 1–42 (2021).

23. Yassour, M. *et al.* Strain-Level Analysis of Mother-to-Child Bacterial Transmission during the First Few Months of Life. *Cell Host Microbe* **24**, 146–154 (2018).

24. DIABIMMUNE. DIABIMMUNE Study Metagenomic Data. Available at: https://diabimmune.broadinstitute.org/diabimmune/data/14/. (Accessed: 15th January 2021)

25. Segata, N. *et al.* Metagenomic microbial community profiling using unique clade-specific marker genes. *Nat. Methods* **9**, 811–814 (2012).

26. Kaminski, J. *et al.* High-Specificity Targeted Functional Profiling in Microbial Communities with ShortBRED. *PLOS* **486**, 207–214 (2015).

27. Alcock, B. P. *et al.* CARD 2020: antibiotic resistome surveillance with the comprehensive antibiotic resistance database. *Nucleic Acids Res.* **48**, D517–D525 (2020).

28. Suzek, B. E., Wang, Y., Huang, H., McGarvey, P. B. & Wu, C. H. UniRef clusters: A comprehensive and scalable alternative for improving sequence similarity searches. *Bioinformatics* **31**, 926–932 (2015).

29. UniProt. UniRef90 Download (Last modified October 7, 2020). Available at: https://ftp.uniprot.org/pub/databases/uniprot/uniref/uniref90/. (Accessed: 3rd November 2020)

30. Mallick, H. *et al.* Multivariable Association Discovery in Population-scale Meta-omics

Studies 3. *bioRxiv* 2021.01.20.427420 (2021).

31. Rahnavard, G. *et al.* High-sensitivity pattern discovery in large multi'omic datasets. (2021).

32. Lahti, L. & Sudarshan, S. Tools for microbiome analysis in R. (2017).

33. Caniglia, E. C. & Murray, E. J. Difference-in-Difference in the Time of Cholera: a Gentle Introduction for Epidemiologists. *Curr. Epidemiol. Reports* **7**, 203–211 (2020).

34. Wing, C., Simon, K. & Bello-Gomez, R. A. Designing Difference in Difference Studies: Best Practices for Public Health Policy Research. *Annu. Rev. Public Health* **39**, 453–469 (2018).
